# Long Time Scale Molecular Dynamics Simulation of Magnesium Hydride Dehydrogenation Enabled by Machine Learning Interatomic Potentials

**DOI:** 10.1021/acsaem.4c02627

**Published:** 2024-12-19

**Authors:** Oliver Morrison, Elena Uteva, Gavin S. Walker, David M. Grant, Sanliang Ling

**Affiliations:** † Advanced Materials Research Group, Faculty of Engineering, University of Nottingham, Nottingham NG7 2RD, United Kingdom; ‡ Aria Sustainability Ltd., Unit 7, Wheatcroft Business Park, Landmere Lane, Edwalton, Nottingham NG12 4DG, United Kingdom

**Keywords:** Magnesium Hydride, Molecular Dynamics Simulations, Machine Learning Interatomic Potentials, Hydrogen Storage, Density Functional Theory

## Abstract

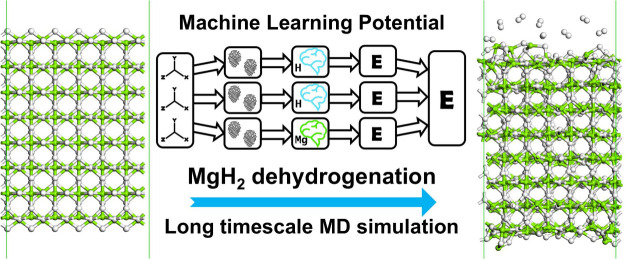

Magnesium hydride (MgH_2_) is a promising material for solid-state hydrogen storage due to its high gravimetric hydrogen capacity as well as the abundance and low cost of magnesium. The material’s limiting factor is the high dehydrogenation temperature (over 300 °C) and sluggish (de)hydrogenation kinetics when no catalyst is present, making it impractical for onboard applications. Catalysts and physical restructuring (e.g., through ball milling) have both shown kinetic improvements, without full theoretical understanding as to why. In this work, we developed a machine learning interatomic potential (MLP) for the Mg–H system, which was used to run long time scale molecular dynamics (MD) simulations of a thick magnesium hydride surface slab for up to 1 ns. Our MLP-based MD simulations reveal previously unreported behavior of subsurface molecular H_2_ formation and subsequent trapping in the subsurface layer of MgH_2_. This hindered diffusion of subsurface H_2_ offers a partial explanation on the slow dehydrogenation kinetics of MgH_2_. The kinetics will be improved if a catalyst obstructs subsurface formation and trapping of H_2_ or if the diffusion of subsurface H_2_ is improved through defects created by physical restructuring.

## 1 Introduction

Hydrogen energy has long been touted as a potential candidate for future green industries.^1,2^ It is the eighth most abundant element on Earth by atomic fraction and has the highest gravimetric energy capacity of any other chemical energy carriers (142 MJ/kg).^3^ Currently, one of the biggest challenges to a widespread hydrogen economy is efficient storage.

At ambient temperature and atmospheric pressure, 1 kg of hydrogen gas has a volume of 11 m^3^ (a density of around 0.09 g/L).^4^ The density of hydrogen gas can be improved through compression, with a pressure of 700 bar achieving a much improved (yet still suboptimal) density of 39.6 g/L at room temperature.^5,6^ However, containing such a high pressure gas requires high-strength pressure vessels and poses safety concerns for on-board storage. Liquid storage achieves a higher density of 70.8 g/L but is significantly hindered by the energy-intensive liquefaction process, ultimately making it a less energy-efficient solution.^7^

Solid-state storage is an alternative route that bypasses these problems by binding the gas through either a reversible hydrogenation reaction or physical adsorption in the material. Magnesium hydride is one of the most studied and promising candidate materials owing to its low production costs, low toxicity, and high gravimetric and volumetric hydrogen-storage capacities of 7.6 wt % and 110 g/L, respectively.^8^ However, the material fails to meet all the physical requirements due to its slow (de)hydrogenation kinetics and high dissociation temperatures; the latter stems from the thermal stability of the hydride, exemplified by a high dehydrogenation enthalpy of Δ*H*_dec_ = 74.06 ± 0.42 kJ/mol H_2_.^9^ At a working temperature of 300 °C and pressure of 30 bar, the rate of hydrogenation ranges from 6 h to 2 weeks, with the high variability due to the immutable kinetics which have been found to be affected by factors such as particle size and material purity.^10,11^

Research has shown that the kinetics can be improved by nanostructuring (such as through ball milling of the metal) with a 10-fold increase in the amount of hydrogen released by magnesium hydride clusters over the span of an hour relative to the bulk metal.^12^ Catalysts have been shown to yield more significant kinetic improvements^13^ with transition metal-based catalysts; e.g., TiH_2_ ball milled with MgH_2_ resulted in the evolution of 6 wt % of hydrogen in as little as 10 min.^14^ Other catalysts reported in the literature include transition metal oxides such as MoO_2_, V_2_O_5_, and Nb_2_O_5_ and transition metal chlorides.^15,16^ Despite the kinetic improvements from nanostructuing and/or catalysts, there are still challenges in a system retaining the initial very fast kinetics. This is a result of factors such as grain growth during cycling, catalyst migration, oxidation, and loss of nanoscale structure.^17^ A lack of understanding about the reaction pathway means that there is no concrete explanation as to the mechanism behind which nanostructuring or catalysts improve the kinetics of dehydrogenation. Consequently, investigations to improve MgH_2_ kinetics still remain subject to time and resource costly trial-and-error approaches rather than rational design based on prior mechanistic knowledge.

Although the hydrogenation reaction pathway has not yet been fully elucidated, it is presumed to entail the following steps: physisorption of molecular hydrogen onto the metal surface through van der Waals interactions, dissociation of molecular hydrogen into hydrogen atoms, formation of chemical bonds between magnesium and hydrogen atoms, and finally, diffusion of hydrogen atoms throughout the material.^4,18^ Nucleation of the hydride phase remains a point of contention, with differing views on whether this initiates at the surface and grows inward or in the bulk and grows outward.^19^

For the optimal design of a catalyzed magnesium hydride material, an atomistic understanding of the reaction pathway is crucial. This insight is challenging to obtain experimentally, making *in silico* molecular dynamics (MD) simulations the preferred approach. The accuracy of any MD simulation is directly determined by the underlying interatomic potential, which maps atomic structures to their corresponding potential energy and forces, guiding the simulation of the dynamic system.

Analytical force fields that describe the potential as closed-form equations motivated by initial assumptions about the physical domain are computationally inexpensive to evaluate but are notoriously inaccurate for complex systems with multiple instances of bond breaking and formation. Force field accuracy is dependent on the accuracy of parametrization, which is a nontrivial task and significantly labor intensive. A ReaxFF potential for magnesium and magnesium hydride systems was constructed by Cheung et al.^20^ and was applied to examine the relationship between grain size and heat of formation. While the force field offers valuable information, MD simulations by Zhou et al.^21^ found that the potential led to an unstable MgH_2_/Mg solid-state interface even at lower temperatures of 300 K. Zhou et al. produced a bond order potential (BOP) for Mg–H systems and five 1 ns MD simulation runs. Although their MD simulations captured relevant (de)hydrogenation chemical reactions, e.g., 2H (gas) → H_2_ (gas) and 2H (gas) + Mg (hcp) → MgH_2_ (rutile), the BOP potential was not parametrized to generalize across the full reaction pathway due to not being optimized for the simulation of the full dehydrogenation reaction, which involves events like atomic diffusion and bond breaking/formation at realistic complex surfaces, which were not considered in their target structures when they parametrized their BOP potential.

*Ab initio* methods, where the potential is calculated from first principles, offer greater accuracy at the expense of computational time. The most frequently used *ab initio* method for solid-state modeling is the mature field of density functional theory (DFT). It offers a good trade-off between accuracy and computational cost and is widely applied in solid-state materials modeling. For example, Dong et al. studied the layer-by-layer dehydrogenation of a MgH_2_(110) surface slab using DFT, finding that the surface H_2_ desorption has the highest energy barrier of 2.5 eV in MgH_2_ dehydrogenation, because the H vacancy formed after dehydrogenation in the surface layer has a high electron localization.^22^ However, even with the improved trade-off, DFT quickly becomes too computationally costly when simulating larger systems containing hundreds of atoms or running simulations over millions of time steps, as required to span the full dehydrogenation period.

A possible solution is the use of machine learning interatomic potentials. Machine learning (ML) is a branch of artificial intelligence that discovers underlying patterns in data without explicit instructions and is often hailed as the fourth scientific revolution. ML is growing in prominence in computational modeling as it offers a unique solution to producing force-configuration space mappings. Rather than using mathematical closed-form expressions based on physical domain knowledge, the relationship is learned by using a small training set of data to update model parameters through optimization of a loss function. Once a desired level of accuracy is obtained, the trained ML model is used as an emulator of the electronic structure calculator at a fraction of the computational cost. Out of all of the machine learning methods, artificial neural networks (ANNs) are now the most widely applied to model potentials due to their ability to handle large data sets and act as universal approximators that can in principle model any function to arbitrary precision. They also benefit from the ease of application thanks to open-access frameworks like PyTorch^23^ and TensorFlow^24^ that allow for modular neural network construction.

There is existing research that performs MD simulations of Mg–H systems using *ab initio* methods, but these have been restricted to a time scale of tens of ps and have therefore only partially captured the dehydrogenation, providing insight only into specific aspects of the process.^25^ A machine learning interatomic potential (MLP) has previously been constructed by Wang et al. with the primary goal of examining the behavior of magnesium hydride clusters.^26^ Wang et al. achieved a test-set RMSE for energy and force of 31.25 and 189.9 meV/Å, respectively. They have used a large data set of 22,965 reference data points, 90% of which were seen by the model as the training set and 10% was used as a validation set. Although their potential was not intended for the study of surface reactions, the adaptive sampling method from high-temperature MD simulation runs meant there were data points for breaking molecular H–H bonds that made it possible to model the H_2_ dissociation on a surface.

The work reported below is distinguished by several key factors. It simulates MgH_2_ over long time scale (up to 1 ns) which captures the full atomistic details for the initial stage of MgH_2_ dehydrogenation. The model achieves high accuracy despite using a training set of only a few hundred structures. By employing an active learning approach, the effectiveness of the training was enhanced, mitigating the use of a small training set and allowing for faster training and more efficient model refinement. Additionally, while most MLP research focuses on realistic structures, this work deliberately includes extreme, unrealistic configurations in the training set. These structures help the model better simulate real-world phenomena by teaching it to recognize and avoid such unrealistic configurations.

## 2 Methodology

### 2.1 Model Construction

**Figure 1:**
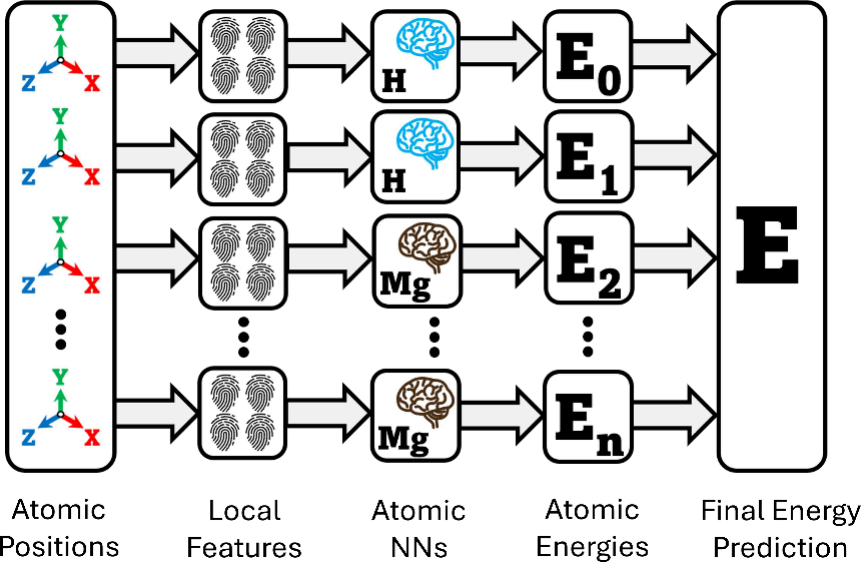
Schematic of the BPNNP. The model consists of multiple neural networks, each responsible for a unique element. Atom centred symmetry functions (ASCFs) calculate descriptors for each local environment. Descriptors are processed by corresponding ANN to predict atomic energy contribution. All atomic energy contributions are summed up to calculate total energy.

Artificial Neural Networks were employed based around the Behler-Parinello Neural Network Potential (BPNNP)^27^ (see Figure 1), which enabled application of ANNs to handle scalable multidimensional potential energy surfaces by representing the system energy as a sum of local atomic energy contributions.

Atomic coordinates were converted to symmetry functions, which encoded the local chemical environment of each atom into a descriptor that is invariant to translation, rotation, and exchange of identical atoms. The radial symmetry function is expressed as
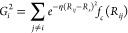


A cosine cutoff function, *f*_*c*_(*R*_*ij*_), is used to ensure smooth decay after the cutoff, preventing functional discontinuities:
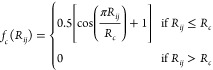
where *R*_*ij*_ is the distance between particles *i* and *j*, *R*_*c*_ is the cutoff distance.

The angular symmetry function is given by
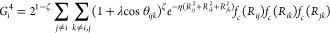
where *i*, *j*, *k* are indices representing atoms, with *i* as the central atom and *j*, *k* as neighboring atoms. *R*_*ij*_, *R*_*ik*_, and *R*_*jk*_ denote the distances between pairs of atoms: *R*_*ij*_ is the distance between atoms *i* and *j*, *R*_*ik*_ is the distance between atoms *i* and *k*, and *R*_*jk*_ is the distance between atoms *j* and *k*. The angle θ_*ijk*_ is the angle formed by central atom *i* and its neighbors *j* and *k*.

Committee Neural Network Potential (C-NNP)^28^ was used, where an ensemble of multiple models trained on slightly different sets was used to obtain a cumulative prediction average over energy and force values. It was found that a single NNP model displayed nonphysical behavior in the test MD runs, but this behavior was mitigated when increasing the number of NNP models in a C-NNP model. A committee of 8 models was chosen to be the optimal size as ensembles with a higher number of models did not yield any consistent improvement in accuracy or transferability. The C-NNP model was trained to predict energies relative to atomic reference energies, and it used 20 radial symmetry functions and 60 angular symmetry functions.

Predictions were more accurate when trained to predict energies relative to atomic reference energies, which correspond to DFT energy predictions for optimized bulk Mg and H_2_ gas structures divided by atom count. The C-NNP model was trained on relative DFT energy values. After training, reference energies can be added to each of the model’s energy predictions to give absolute energy. In this instance, this subsequent correction is not necessary, as this work only uses the C-NNP model to produce simulations with constant hydrogen and magnesium counts. In such simulations, the reference energy is held constant and does not affect the calculation of forces as forces depend on changes to the energy, not the energy values themselves. As using relative energies has no bearing on the results, the raw values returned by C-NNP models are presented here as “potential energies” without conversion to absolute energy.

Model training was iterated over epochs until a convergence threshold was achieved. A training convergence threshold was set for an energy root-mean-square error (RMSE) of ξ_0_ = 0.1 meV/atom and for the highest energy error of ξ_0_ = 1 meV/atom. After training completion, the model with the best validation set error was selected as the final model.

**Figure 2:**
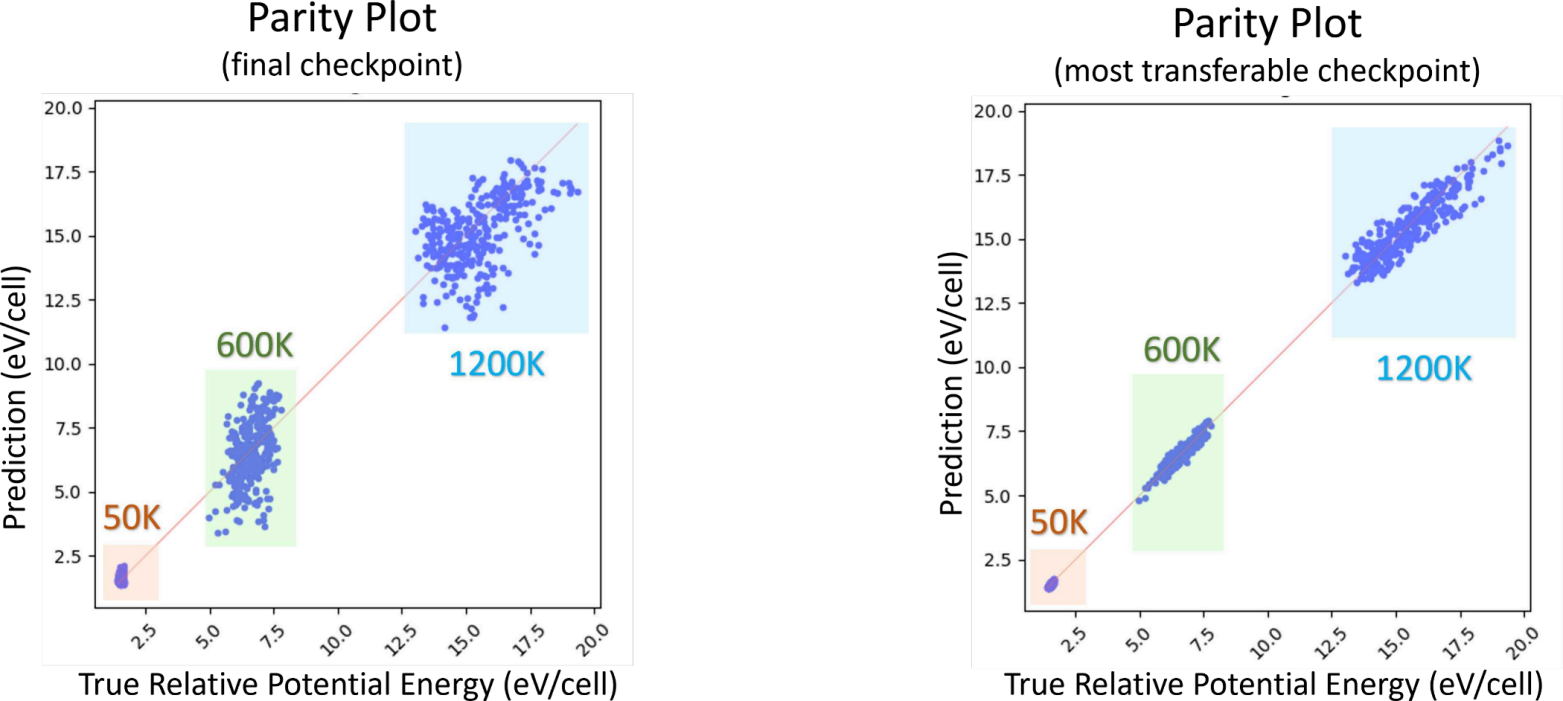
Parity graphs showing the respective performance of two checkpoints produced during the training process of a NNP. Validation was performed on selected structures from the trajectories of *ab initio* molecular dynamics (AIMD) simulations at three different temperatures, which have been highlighted in different colors.

Overfitting of the results was mitigated through the addition of a validation step during the training protocol. The training data set was split into 90% seen and 10% unseen data, and the chosen parameters were selected on the basis of the best validation error. Figure 2 shows the parity plots between the predicted energies and the actual DFT calculations. Selecting final parameters based on lowest validation error outperformed a selection based on the final training epoch, having respective potential energy test errors of 4.78 and 13.81 meV/atom on unseen data. It also demonstrated good model transferability as the test error was close to the seen data RMSE of 4.27 meV/atom, signifying that the model was not just biased toward the training set but was able to generalize effectively to previously unexplored configurations.

The final model was trained on 316 structures and achieved an RMSE of 23.4 meV/atom and 56.56 meV/Å for energy and force, respectively, tested on unseen data.

### 2.2 Training Data

Since MLPs derive patterns from training data, the generalizability of an MLP is dependent on a representative sample of the total configuration space to maximize information learned in the training process and provide good transferability.

Reference data were generated from geometrical optimizations and molecular dynamics simulations between the ranges of 50 and 1500 K for a wide range of Mg–H structures, such as bulk Mg and MgH_2_, H_2_ gas, MgH_2_ surface slab, H_2_ gas on Mg surface slab, Mg/MgH_2_ interface, Mg and Mg–H clusters, etc. (see Supporting Information). Calculations were performed using CP2K^29^ at DFT level with a mixed plane wave/Gaussian basis set.^30^ A Gradient Generalized Approximation (GGA) correlation function was utilized with the Perdew–Burke–Ernzerhof variant^31^ with Grimme’s D3 dispersion correction (PBE+D3) including the C9 term.^32^ Double-ζ polarization quality Gaussian basis sets^33^ and a 400 Ry plane-wave cutoff for the auxiliary grid were employed, in conjunction with the Goedecker–Teter–Hutter pseudopotentials.^34,35^ For self-consistent field (SCF) calculations, a target accuracy of 1 × 10^–6^ Hartree was used. All structures bar Mg systems that were produced using K-point sampling were employed using large cell sizes with periodic boundary conditions and Γ-point approximation.

A total of 62,471 reference structures were generated, via DFT-based MD simulations and geometry optimizations, which served as a Mg–H reference data set for the development of Mg–H potentials. Physically unusual structures (labeled through single-point DFT calculations) were added to the training set by either manually constructing structures to replicate unwanted behaviors (such as three hydrogen atoms forming a H_3_ molecule) or by randomly perturbing all the atoms in a reference structure. Instances of extreme bonding were required to steer the MD simulation away from nonphysical behavior.

A small subset of the reference data was selected as the final training set by filtering the full data set through a “Query by Committee (QbC) process”, using Atomic Machine Learning software by Marsalek et al. with slight modifications to handle structures with different cell sizes.^28^ This process takes advantage of the C-NNPs capability to quantify the uncertainty of a prediction. QbC uses uncertainty to identify the structures that are most valuable to the training set, enabling an actively diverse selection of training data.

**Figure 3:**
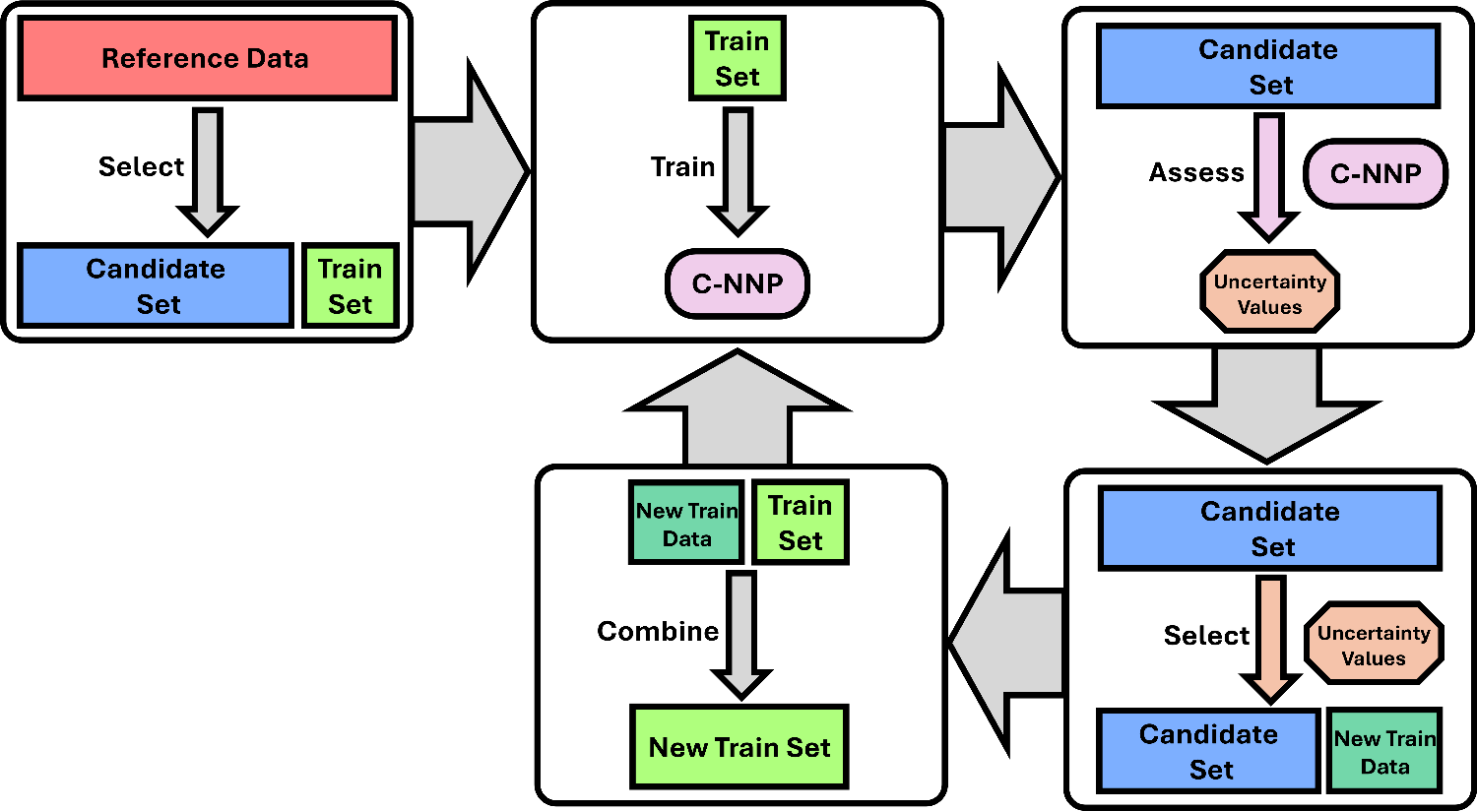
Visualization of the QbC process.

As visualized in Figure 3, the process of QbC is divided into the following five steps:

- Step A: A random selection of structures is sampled from the reference data. These reference structures form a small initial training set. The remaining structures (those that were not selected) form a new set called the candidate set.
- Step B: The training set is used to train a C-NNP.
- Step C: The C-NNP assigns an uncertainty value to each candidate structure.
- Step D: The candidate structure with the highest uncertainty values are deemed to contain the most unique information. These structures are removed from the candidate set.
- Step E: The high uncertainty structures that were removed from the candidate set are added to the training set.

If the resulting training set is sufficiently large, then the process is complete, and the new training set can be used to train a final model. Alternatively, steps B–E can be repeated until the training set reaches the desired size.

When a structure is selected to join the training set, this is indicative of information contained in that structure that is not captured well by the existing training points. This means that the resulting training points share as little information as possible. QbC ensured the C-NNP model was trained in a reasonable time frame by reducing the size of the training set (e.g., by removing structures which do not contribute much to the training results), while simultaneously achieving more accurate and transferable results.

### 2.3 Software

The Atomic Simulation Environment (ASE)^36^ was used for data management and analysis. AML,^28^ interfaced with n2p2,^37^ which implements BPNNP, was used for the QbC process and C-NNP training and testing. DFT- and C-NNP-based MD simulations were performed using CP2K,^29,30^ with results visualized through Visual Molecular Dynamics (VMD)^38^ and Materials Studio.^39^

### 2.4 Molecular Dynamics Simulations

Slabs were constructed consisting of the most stable α-MgH_2_ polymorph with thickness varying between 2 and 16 Mg layers; for production runs and subsequent analysis, this work focused on a surface slab model with 8 Mg layers. Periodic cells were expanded by >100 Å in the direction perpendicular to the surface slab to provide a sufficiently large vacant space for the released hydrogen gas molecules, with the most stable surface (110) exposed to the vacant space.^40,41^ DFT (PBE + D3) geometry optimizations were performed prior to carrying out MD simulations to ensure slabs were physically realistic and stable. MD simulations were performed using the canonical ensemble (NVT) with a 0.5 fs time step and were run for 1 ns (2,000,000 timesteps) at a temperature of 800 K, which is slightly higher than the experimentally observed dehydrogenation temperature of MgH_2_, in order to accelerate the rate of dehydrogenation reaction, so more events of H_2_ desorption can be observed from the C-NNP-based MD simulations for subsequent analysis. To maintain the temperature of a simulation, a Canonical Sampling through Velocity Rescaling (CSVR) thermostat^42^ was used to maintain the temperature, using a time constant of 50 fs (for DFT-based MD) or 100 fs (for C-NNP-based MD). For C-NNP-based MD simulations, the trajectory was saved every 10 fs (20 MD timesteps), to obtain more precise information (e.g., timing of key events such as H_2_ formation and desorption from the surface) for further analysis.

## 3 Results and Discussion

### 3.1 Further Model Validation

To judge the accuracy of force and energy predictions, the model was used to run 36 MD simulations at different temperatures for a variety of Mg–H structures that are similar to those considered in the training set but are much smaller than the thick MgH_2_(110) surface slab considered for production MD simulations based on the best C-NNP model. These simulations each ran for 1 ns with a 0.5 fs time step. After 200 ps of simulation time, single-point energy and force calculations were performed using both the DFT method and the C-NNP model every 100 ps. The two sets of results were compared to assess the model’s performance.

**Figure 4:**
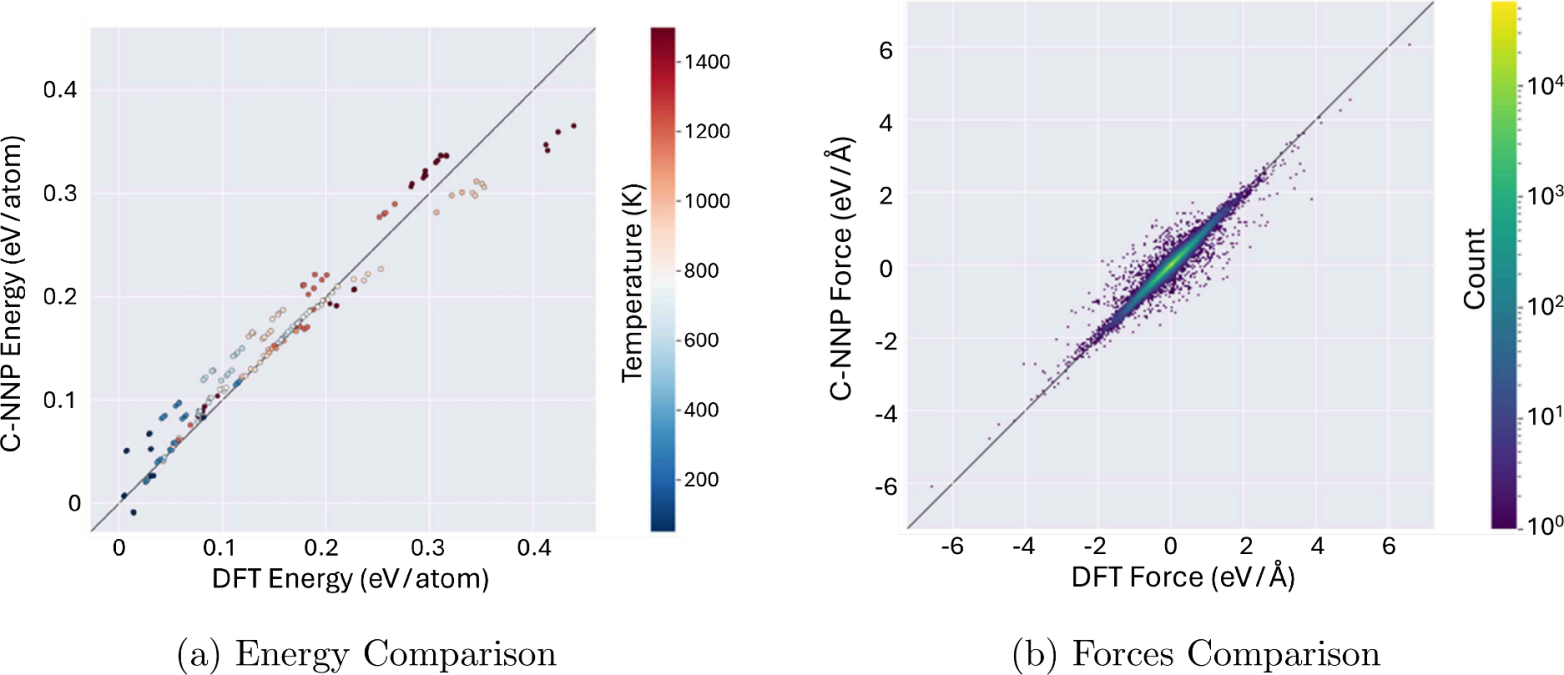
Parity graphs, comparing the predictions of the C-NNP model with the predictions of DFT on (a) energy and (b) forces.

As shown in Figure 4, the model made accurate predictions, with an RMSE of 23.4 and 56.5 meV/Å for energy and force, respectively. For comparison, Wang et al. trained and tested a MLP for Mg–H system and achieved an RMSE performance of 31.25 meV/atom and 189.9 meV/Å for energy and force, respectively.^26^ Given the wide range of temperatures and variety of Mg/H coordination environments in the validation set, these low prediction errors give reassuring indication that the C-NNP model can maintain stable performance over long time scale simulations.

### 3.2 Hydrogen Molecule Formation

**Figure 5:**
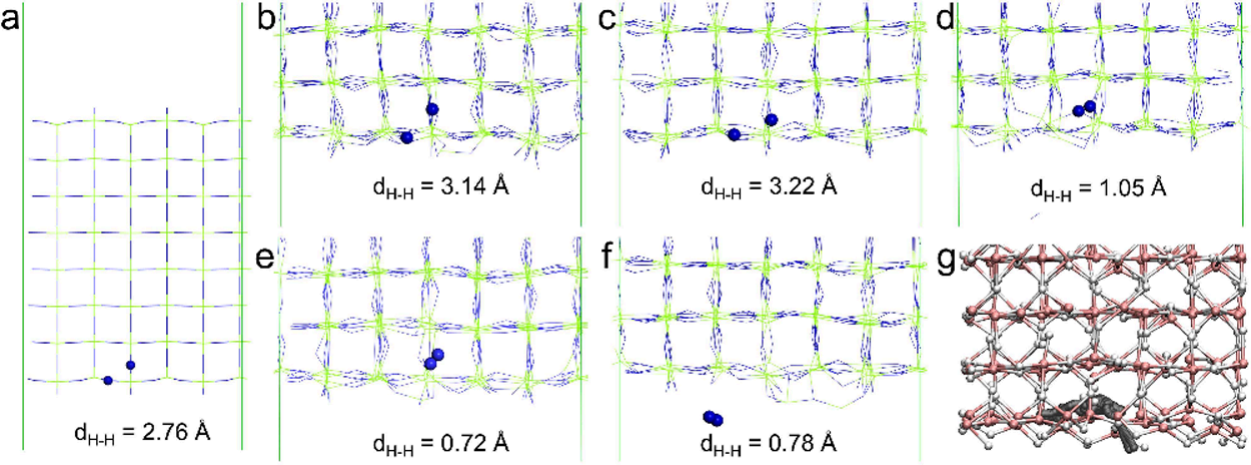
(a) An 8-layer thick MgH_2_(110) surface slab in a 6 × 3 supercell. The two H atoms of interest are represented as blue balls. Atomic structure of the MgH_2_ surface slab at MD simulation timesof (b) 304.62 ps, (c) 305.62 ps, (d) 306.62 ps, (e) 307.62 ps, and (f) 349.58 ps. (g) Trail of the two H atoms of interest (gray balls) from the onset of formation utill its release from the surface slab. Mg and H atoms are represented in green and blue sticks in (a)–(f), respectively. Mg and H atoms are represented in pink and white balls in (g), respectively. For clarity, only part of the surface slab is shown in (b)–(g).

Using the most transferable C-NNP, a long time scale and large-size molecular dynamics simulation was run, aiming to get a better fundamental understanding of the dehydrogenation mechanism of MgH_2_. Previous DFT calculations have shown that the (110) surface is the most stable surface facet of MgH_2_,^40,41^ so a MgH_2_(110) surface slab with an 8-layer thickness in a 6 × 3 supercell (a total of 864 atoms, see Figure 5a) was considered in the MD simulation. The MD simulation was run for a duration of 1 ns with an MD time step of 0.5 fs (a total of 2,000,000 MD steps) at a temperature of 800 K. Despite the fact that the MD simulation was run for such a long period of time, we only observed the desorption of eight hydrogen molecules in this simulation, corresponding to <3% of the total number of hydrogen atoms in the surface slab.

Next, the individual process for the formation and desorption of the eight hydrogen molecules was investigated. It is generally believed that the desorption of H_2_ molecules from MgH_2_ follows the process of hydrogen atoms diffusing from the bulk (or subsurface) toward the surface of MgH_2_, after which two hydrogen atoms combine at the surface to form a H_2_ molecule which then desorbs from the surface. Interestingly, it was found that all eight H_2_ molecules that desorbed within the 1 ns MD simulation were not formed on the surface; instead, they were formed in the subsurface layer (defined as the layer with a distance of <5 Å into the bulk from Mg atoms on the surface with 5-fold coordination) of the MgH_2_(110) surface slab and diffused to the surface.

Taking one of the eight desorbed H_2_ molecules (Molecule 3) as an example, the atomic structures of these two H atoms involved in Molecule 3 at various stages are shown in Figure 5. At the start of the simulation (a pristine MgH_2_(110) surface slab, see Figure 5a), one H atom was located on the surface, with the other H atom located between the top layer and subsurface layer, and the two H atoms were separated by a distance of 2.76 Å (Figure 5a). During the next 300 ps of MD simulation, the two H atoms vibrated around their equilibrium positions: at 304.62 ps, the two H atoms were separated by a distance of 3.14 Å (Figure 5b), and at 305.62 ps, the two H atoms were separated by a distance of 3.22 Å (Figure 5c). After another 1 ps, the two H atoms started to vibrate toward each other, with a much shorter distance of 1.05 Å at 306.62 ps (Figure 5d). Immediately after that, a H_2_ molecule was formed in the subsurface layer, and the interatomic distance between the two H atoms oscillated around 0.7 Å (Figure 5e). Interestingly, the H_2_ molecule formed in the subsurface layer did not desorb from the surface immediately. Instead, it was trapped in the subsurface layer for another 43 ps before it was able to diffuse to the surface from where it was immediately desorbed (Figure 5f). The trail of this H_2_ molecule (represented as gray balls) from the onset of formation in the subsurface layer until its release from the surface slab is shown in Figure 5g. It was found that this H_2_ molecule moved around near its initial site of formation before it was desorbed from the surface.

**Figure 6:**
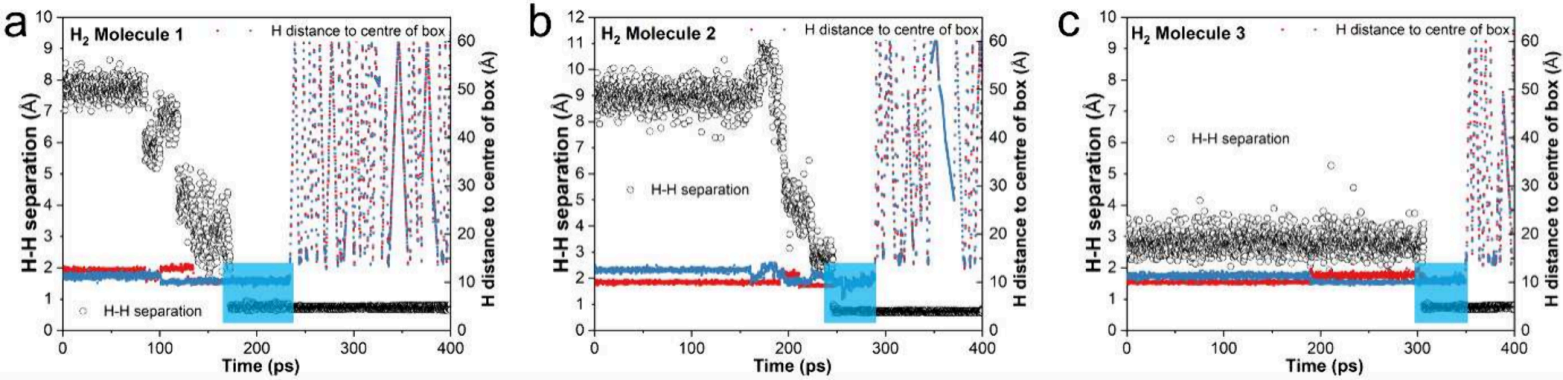
Interatomic distances (left *Y*-axis, represented as empty circles) between H atoms and their location (relative to the center of the simulation box, right *Y*-axis, represented as red and blue dots) of three selected H_2_ molecules as a function of MD simulation time (*X*-axis): (a) Molecule 1, (b) Molecule 2, and (c) Molecule 3. The trapped H_2_ molecules are indicated by light blue shaded areas on the plots.

After analysis of the desorption process of the other seven H_2_ molecules, it was found that many other H_2_ molecules were also trapped in the subsurface layer before they were released. Among the eight H_2_ molecules desorbed within the 1 ns MD simulation, six of the H_2_ molecules were trapped in the subsurface layer for more than 10 ps before release, with one H_2_ molecule being trapped for 61 ps. Only two H_2_ molecules desorbed from the surface within 1 ps of formation in the subsurface layer. Further analysis of the three H_2_ molecules which were trapped in the subsurface layer for more than 40 ps, including Molecule 3 discussed earlier, is shown in Figure 6. While the two H atoms involved in the formation of Molecule 3 were initially located next to each other with a separation of 2.76 Å (Figures 5a and 6c), it was found that this was not the case for Molecules 1 and 2, of which the initial H–H separations at the start of the MD simulation were 7.71 Å (Figure 6a) and 8.95 Å (Figure 6b), respectively. In fact, it was found that among the eight H_2_ molecules desorbed, there were only two H_2_ molecules of which the initial H–H separations are smaller than 3.9 Å; for the other six H_2_ molecules, the initial H–H separations range between 6.9 and 9.4 Å, indicating atomic diffusion of H atoms (see Figure 6a and b) was needed before the two H atoms can combine to form a H_2_ molecule. For the locations (relative to the center of the simulation box) of hydrogen atoms in Figure 6, it was also found that the three H_2_ molecules were indeed trapped in the subsurface layer for a period of time (between 43 and 61 ps, represented by short H–H separation and nearly constant distance of 10 Å to the center of the box), before they desorbed from the surface (exemplified by large variations in the distance of H atoms to the center of simulation box). Checking the origin of the eight desorbed H_2_ molecules, it was found that H atoms involved in the formation of these eight H_2_ molecules were initially located at the surface or subsurface. While it was found from the 1 ns MD simulation that H_2_ molecules only formed in the subsurface layer, it is plausible that, e.g., if the MD simulation is run for much longer, H_2_ molecules may also form deeper in the surface slab, or that H atoms may diffuse from deep within the slab to the surface or subsurface and combine with other H atoms to form H_2_ molecules.

**Figure 7:**
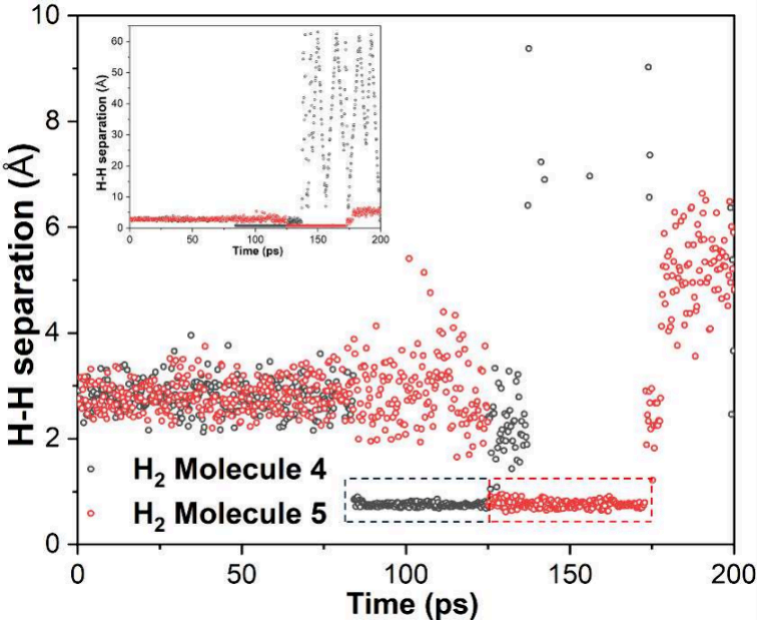
Interatomic distances (*Y*-axis, represented as empty circles) between H atoms of two selected H_2_ molecules as a function of MD simulation time (*X*-axis). The H–H distances related to the two H_2_ molecules are represented by black and red circles, respectively. The temporarily formed H_2_ molecules (represented by small H–H separations) are indicated by dashed boxes on the plots. The inset figure shows the same data but with full scale *Y*-axis, showing the large H–H separation for the two H atoms involved in Molecule 4 after the H_2_ molecule dissociation at 125 ps.

In addition to the eight H_2_ molecules which were initially formed in the subsurface layer and ultimately desorbed from the surface, there were two incidences where H_2_ molecules were formed in the subsurface layer of the slab and were trapped in the interior for a period of time (41 and 48 ps, respectively), before the H_2_ molecules dissociated into atomic H again, see Figure 7. In fact, one of the H atoms (Atom 112) involved in the formation of Molecule 1 initially formed an H_2_ molecule (Molecule 5) with a different H atom (Atom 133) and was trapped in the subsurface layer for about 48 ps (see Figure 7), after which the H_2_ molecule (Molecule 5) dissociated. Interestingly, before the formation of Molecule 5 at around 125 ps, Atom 133 formed another H_2_ molecule (Molecule 4) with a third hydrogen atom (Atom 284) at around 84 ps, which was trapped in the subsurface layer for about 41 ps (see Figure 7), after which the H_2_ molecule (Molecule 4) dissociated, with Atom 133 forming a new H_2_ molecule (Molecule 5, see Figure 7) with Atom 112 shortly after, and Atom 284 forming a H_2_ molecule with a different H atom at around 125 ps which desorbed from the surface at around 137 ps (i.e., this H_2_ molecule was trapped for about 12 ps). This is the reason why the H–H separation between Atom 133 and Atom 284, which were involved in the formation of Molecule 1, became so big after about 137 ps (see the inset of Figure 7).

### 3.3 Discussion

As shown in the Results and Discussion, it was found that the formation of H_2_ molecules in the subsurface layer during the initial stage of the dehydrogenation of MgH_2_ is common. For all eight H_2_ molecules desorbed within an MD simulation time of 1 ns at 800 K, all of them were initially formed in the subsurface layer, and the majority of them (6 out of 8) were trapped in the subsurface layer for more than 10 ps before they were released from the surface slab, with one H_2_ molecule trapped for more than 60 ps before desorption. It was also found that H_2_ molecules can form and get trapped (e.g., for more than 40 ps) in the subsurface layer temporarily before they dissociated, followed by atomic hydrogen diffusion into vacancy or formation of new H_2_ molecules with other hydrogen atoms nearby. These simulation results provide new insights into the atomic mechanism of the dehydrogenation of MgH_2_. It is well-known that the kinetics of the MgH_2_ dehydrogenation reaction is sluggish. For example, Hanada et al. showed that it took more than 200 min at 308 °C to fully dehydrogenate pure ball-milled MgH_2_.^43^ Long time scale MD simulation based on the C-NNP indicates this could be partly due to the formation and trapping of H_2_ molecules in the subsurface layer of MgH_2_. The MLP based MD simulation was run at a relatively high temperature of 800 K in order to accelerate the rate of the dehydrogenation reaction so more H_2_ desorption events can be observed. The trapping time of molecular H_2_ in the subsurface layer of MgH_2_ slab as observed in the MD simulation is likely to be much longer, if the temperature of the MD simulation is decreased, e.g. to 581 K (the experimentally observed dehydrogenation temperature of uncatalysed ball-milled MgH_2_).^43^ The long trapping time of molecular H_2_ in the subsurface layer may play an important role in the sluggish dehydrogenation kinetics of uncatalysed MgH_2_.

**Figure 8:**
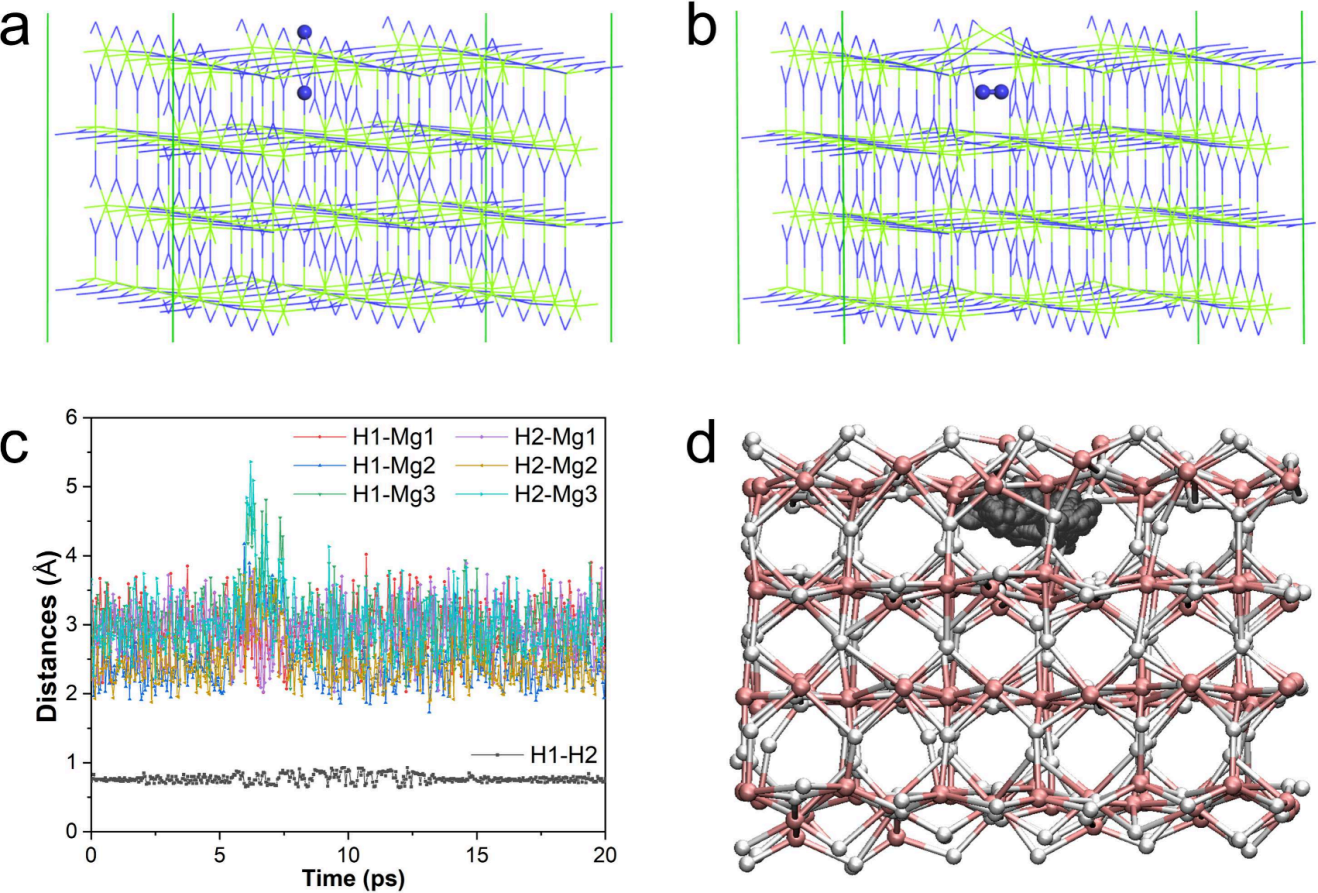
DFT optimized structures of a 4-layer thick MgH_2_(110) surface slab in a 6 × 3 supercell: (a) the perfect cell and (b) a manually created cell with a molecular H_2_ being trapped in the subsurface layer. The two H atoms of interest are represented as blue balls. (c) Distances between the two H atoms of interest (H1 and H2) and between the two H atoms and three nearby Mg atoms (Mg1, Mg2, and Mg3) as a function of MD simulation time. (d) Trail of the two H atoms of interest (gray balls) during the DFT-based MD simulation. Mg and H atoms are represented in green and blue sticks in (a) and (b), respectively. Mg and H atoms are represented in pink and white balls in (d), respectively.

To verify that the formation of the H_2_ molecules in the subsurface layer of MgH_2_ was not an artifact of the C-NNP, configurations with a H_2_ molecule trapped in bulk MgH_2_ or in the subsurface layer of a MgH_2_(110) slab (with 4-layer thickness, see Figure 8a) were created, by manually moving one H atom toward another H atom to form a trapped H_2_ molecule in the space previously occupied by the second H atom (which produced a H vacancy at the location of the first H atom at the same time), see Figure 8b. The structures were then relaxed using DFT, and the energies of the perfect MgH_2_ configurations and the configurations with trapped H_2_ molecule were calculated using C-NNP. For bulk MgH_2_, it was found the energy penalty of forming a trapped H_2_ molecule was 215 kJ/mol with DFT and 225 kJ/mol with C-NNP (representing an error of <5% and showing excellent accuracy of the C-NNP). In the surface slab, it was found the energy penalty of forming a trapped H_2_ molecule was reduced by more than 25% to 159 kJ/mol with DFT (163 kJ/mol with C-NNP, again, excellent agreement with DFT), possibly due to the lower energy penalty of H vacancy formation at/near the surface and also because Mg atoms have more space to relax near the surface (and less strain), i.e. the energy penalty to displace Mg atoms due to trapping of H_2_ molecule is significantly reduced.

To further verify that the H_2_ molecule is kinetically stable in the subsurface layer of MgH_2_ and is not an artifact of the C-NNP, a DFT-based MD simulation of a 4-layer thick MgH_2_(110) slab with a H_2_ molecule in the subsurface layer (see Figure 8b) was performed for up to 20 ps (40,000 MD timesteps) at a temperature of 800 K. It was found that the trapped H_2_ molecule remained stable throughout the duration of the 20 ps DFT based MD simulation, exemplified by the short H–H distance (below 1 Å) and long Mg–H distances (largely above 2 Å) between the two H atoms and three nearby Mg atoms, see Figure 8c. In Figure 8d, the trail of the two H atoms of interest throughout the duration of the DFT-based MD simulation is shown, and it was found that the trapped H_2_ molecule moved around its immediate vicinity but remained stable without splitting into atomic H and forming chemical bonds with Mg atoms nearby. The long lifetime (>20 ps) of the H_2_ molecule trapped in the subsurface layer observed in the DFT based MD simulation echoes what was observed in the long time scale C-NNP-based MD simulation at the same temperature, which again demonstrates that H_2_ molecules being trapped in the subsurface layer is not an artifact of the C-NNP.

The formation of “internally” trapped H_2_ has been discussed in other related materials; e.g., Fukumuro et al. suggested that Pd vacancies can be formed at higher H concentration in PdHx, which are filled with H_2_ molecules.^44^ In another study by He et al., the authors also showed that H_2_ molecule trapped in the Pd vacancy is stable with a short separation distance of 0.77 Å, and the energy barrier for dissociating the H_2_ molecule in the Pd vacancy to atomic H located near two tetragonal sites was found to be 0.84 eV, which means it is difficult for the H_2_ molecule to thermally escape from the Pd vacancy once the H_2_ molecule is trapped at a high H concentration.^45^ While the energy penalty of forming a trapped H_2_ molecule in bulk and surface slab of MgH_2_ seems to be high, H_2_ molecules being trapped in the subsurface layer of MgH_2_ slab were observed in long time scale MD simulations and will likely be subjected to high energy barriers for dissociation into atomic H and for diffusion into neighboring sites or onto the surface. This can be reflected by the long period of time the H_2_ molecules were trapped in the subsurface layer, before they diffused onto the surface and desorbed from the surface or dissociated into atomic H; of the ten H_2_ molecules formed during the MD simulation, including eight that desorbed from the surface ultimately and two temporarily formed, eight of them were trapped for more than 11 ps, and five of them were trapped for more than 41 ps.

These results suggest the slow dehydrogenation kinetics of uncatalyzed MgH_2_ may be partly due to the formation and trapping of H_2_ molecules in the subsurface layer of MgH_2_. In the work by Hanada et al., the authors found that for a ball-milled MgH_2_ sample with 2 mol % metallic Ni nanoparticles, 90% of the hydrogen was desorbed within 100 min at a much lower temperature of 163 °C.^43^ The much faster kinetics of this catalyzed MgH_2_ dehydrogenation reaction may be explained by the presence Ni metal in the MgH_2_ sample, of which Ni may mix into MgH_2_ during the ball milling process. It is known that Mg metal is not a good catalyst for the dissociation of H_2_ molecule; e.g., Du et al. reported that the dissociation barrier of hydrogen molecule on a pure Mg(0001) surface was 1.05 eV, and incorporating Ti into Mg(0001) surface reduced the barrier to only 0.103 eV due to the strong interaction between the molecular orbital of hydrogen and the d metal state of Ti.^46^ The incorporation of Ni metal in ball-milled MgH_2_ will have a similar effect; i.e., the presence of Ni metal can help to reduce the energy barrier of dissociating H_2_ molecules trapped in the subsurface layer of MgH_2_ into atomic H, which is likely to reduce the trapping time of H_2_ molecules in the subsurface layer. Meanwhile, it is much easier for atomic H to diffuse toward the surface and combine with other H atoms to form H_2_ molecules on the surface and then desorb from the surface. In addition, the mixing of Ni atoms into MgH_2_ could introduce lattice distortion, which may also help to reduce the trapping time of the H_2_ molecules in the subsurface layer.

## 4 Conclusion

A machine learning interatomic potential was developed for the Mg–H system based on accurate DFT reference data obtained on a diverse range of Mg metal, hydrogen, and Mg–H structures featuring different chemical environments of Mg and H. Using a combination of Query by Committee sampling to filter a large reference data set and an additional manual insertion of physically unusual structures to encourage the simulation to avoid unrealistic scenarios, an information-dense training set of 316 structures was used to train the final MLP model. High quantitative accuracy was achieved, exceeding existing MLP error values for the Mg–H system at a fraction of the training data used by other research groups.

Further validation was performed using the best C-NNP on structures not seen during training, and the results were compared with DFT, which showed excellent accuracy of the committee neural network potential. The C-NNP was then used to run long time scale and large size molecular dynamics simulations for up to 1 ns to study the atomistic mechanism of the initial stage of MgH_2_ dehydrogenation. It was found that H_2_ molecules formed in the subsurface layer of the MgH_2_ surface and were trapped for a long period of time before they desorbed from the surface. These results suggest that the slow kinetics of the MgH_2_ dehydrogenation reaction may be partly associated with the formation and trapping of H_2_ molecules in the subsurface layer of MgH_2_.

These results also provide a new interpretation of the experimental observation of why transition metal catalysts such as Ni help to improve the kinetics of the MgH_2_ dehydrogenation reaction. It is hoped these new insights from the computational simulation can be used to design new catalysts that are cheaper and more efficient to accelerate the dehydrogenation reaction of MgH_2_ and make it more suitable for practical hydrogen storage applications. As for the next step, we plan to run even longer molecular dynamics simulations, e.g., for 50 ns (100,000,000 MD timesteps), to observe and understand the atomistic mechanism of the full dehydrogenation of MgH_2_, from which we aim to gain a better understanding of the origin of the sigmoid shape of uncatalysed MgH_2_ dehydrogenation kinetics curve.^43^

## Supporting Information Available

Summary of different structure types considered in the full DFT reference data set of 62,471 structures and corresponding structure count and Mg/H atom count for each structure type (PDF)
