# Supplementary material for: Long Time Scale Molecular Dynamics Simulation of Magnesium Hydride Dehydrogenation Enabled by Machine Learning Interatomic Potentials
Source: ACS Appl Energy Mater. 2024 Dec 19;8(1):492–502. doi: 10.1021/acsaem.4c02627 (PMC11733948; doi:10.1021/acsaem.4c02627)
Supplement: Supplementary file 1 — ae4c02627_si_001.pdf [file ae4c02627_si_001.pdf]

Supporting Information

Long Time Scale Molecular Dynamics Simulation of Magnesium Hydride Dehydrogenation Enabled by Machine Learning Interatomic Potentials

Oliver Morrison[1], Elena Uteva[1], Gavin S. Walker[2], David M. Grant[1], and Sanliang Ling[1,*]

[1]*Advanced Materials Research Group, Faculty of Engineering, University of Nottingham, Nottingham, NG7 2RD, UK*
[2]*Aria Sustainability Ltd, Unit 7 Wheatcroft Business Park, Landmere Lane, Edwalton, Nottingham, NG12 4DG, UK*

***Email: Sanliang.Ling@nottingham.ac.uk**

Structure Type	Structure Count	Mg Count	H Count
**Bulk Mg:**			
HCP MD	721	207,648	0
FCC MD	301	86,688	0
BCC MD	301	86,688	0
HCP MD (vol +20%)	516	148,608	0
HCP MD (vol +40%)	324	93,312	0
HCP MD (vol -20%)	401	115,488	0
HCP MD (vol -40%)	389	112,032	0
**Diatomic:**			
H - H	28	0	56
Mg - Mg	20	40	0
**H_2 Gas:**			
MD 50K	2,201	0	563,456
MD 150K	2,201	0	563,456
MD 300K	497	0	127,232
**H_2 Gas and Mg Slab:**			
MD 50K	2,201	211,296	352,160
MD 600K	2,201	211,296	352,160
MD 1200K	2,401	230,496	384,160
**Rutile MgH_2:**			
MD 50K	1,101	211,392	422,784
MD 300K	1,101	211,392	422,784
MD 600K	1,101	211,392	422,784
MD 900K	1,101	211,392	422,784
MD 1200K	1,101	211,392	422,784
MD 1800K	411	78,912	157,824
**MgH_2 (110) Surface Slab:**			
MD 50K	2,201	316,944	629,486
MD 300K	2,201	316,944	629,486
MD 600K	14,648	2,109,312	4,189,328
MD 900K	2,201	316,944	629,486
MD 1200K	1,301	187344	372,086
**Mg-MgH_2 Interface:**			
MD 50K	2,201	633,888	633,888
MD 600K	1,201	345,888	345,888
MD 1200K	1,201	345,888	345,888
**Meta Stable MgH_2:**			
PA-3 MD	301	32,508	65,016
PBC21 MD	301	32,508	65,016
PBCA MD	301	43,344	86,688
PBCN MD	501	70,140	144,288
**Mg-H Cluster:**			
Mg_{20}	6,120	122,400	0
$Mg_{20}H_{20}$	4,020	80,400	74,660
$Mg_{20}H_{40}$	3,733	74,660	149,320
**Total**	**62,471**	**7,668,576**	**12,974,948**

Table S1: Summary of the different structure types and atom counts in the total reference data set.
